# Environmental health literacy and household air pollution-associated symptoms in Kenya: a cross-sectional study

**DOI:** 10.1186/s12940-020-00643-5

**Published:** 2020-08-25

**Authors:** Jill Raufman, Deanna Blansky, David W. Lounsbury, Esther Wairimu Mwangi, Qing Lan, Jordi Olloquequi, H. Dean Hosgood

**Affiliations:** 1 Global Health Center, Albert Einstein College of Medicine, Bronx, NY USA; 2 Department of Epidemiology and Population Health, Albert Einstein College of Medicine, 1300 Morris Park Ave., Bronx, New York, 10461 USA; 3 The Aga Khan University, Nairobi, Kenya; 4 Division of Cancer Epidemiology & Genetics, National Cancer Institute, National Institutes of Health, Rockville, MD USA; 5 Laboratory of Cellular and Molecular Pathology, Instituto de Ciencias Biomedicas, Universidad Autonoma de Chile, Talca, Chile

**Keywords:** Solid fuel, Household air pollution, Health literacy, Environmental health literacy

## Abstract

**Background:**

Household air pollution (HAP) is a significant source of the global burden of disease. Our objective was to evaluate the association between environmental health literacy (EHL), a domain of health literacy (HL) that describes the ability to use environmental health information to reduce health risks, and symptoms associated with HAP.

**Methods:**

We performed a cross-sectional population-based study of 353 households in Kasarani, Kenya. One individual from each household was surveyed using our novel EHL survey tool. Baseline characteristics were compared between individuals who were symptomatic (i.e., experiencing cough, shortness of breath, phlegm production, wheeze, chest tightness, headache, eye irritation, or burns from cooking at least 5 times per month) versus individuals who were asymptomatic (i.e., experiencing none or symptoms no more than once per month). Multivariate logistic regression was used to determine the odds ratios (OR) of self-reported symptoms associated with HL, stratified by median EHL, adjusting for education, self-perceived health and solid fuel use.

**Results:**

A total of 100 individuals (28%) reported experiencing one or more symptoms at least 5 times per month, including 31.2% of solid fuel users and 30.3% of non-solid fuel users. Among individuals with high EHL, higher HL was associated with lower risk of experiencing symptoms (OR = 0.26; 95% CI 0.10–0.67), however, there was no association among individuals with low EHL (OR = 0.85; 95% CI 0.34–2.13). Among solid fuel users, the association between HL and risk of experiencing symptoms was driven by individuals with high EHL (OR = 0.30; 95% CI 0.05–1.84), rather than those with low EHL (OR = 1.22; 95% CI 0.36–4.16).

**Conclusions:**

To the best of our knowledge, this was the first study to assess the association between EHL, HL, and HAP-associated symptoms. Our findings highlight the potential importance of EHL in promoting sustainable interventions to reduce symptoms associated with HAP from solid fuel use among communities in Kenya.

## Background

Household air pollution (HAP) attributed to the burning of solid fuels, such as coal, wood, and dung for heating and cooking is a key contributor to ambient air pollution, morbidity, and mortality [1, 2]. Worldwide, HAP ranks among the top 10 risk factors for mortality; among countries considered lower income countries (LIC), it ranks as the second leading mortality risk factor [3]. Approximately 3 billion people are exposed to HAP, contributing to a total of over 4 million deaths per year and more than 50% of premature deaths due to pneumonia among children under 5. In Kenya, use of solid fuels for cooking account for 95% of the rural population, 51% of the urban population, and 84% of the national total population, leading to approximately 44,000 deaths per year [4].

Persons living in poverty experience the greatest morbidity and mortality attributable to pollution, with deaths due to HAP highly concentrated in the world’s poorest countries [5], where health literacy (HL) levels are also among the lowest [6]. Within the past 10 years, advances in the production of cheaper, cleaner, improved cookstoves have served to reduce HAP [7]. However, acceptance and adoption of these improved stoves is challenging [8–10]. Although there have been interventions to reduce exposure via safer cooking practices, build community awareness of HAP danger, alter fuel availability, reduce the cost of cleaner cookstoves, improve the actual environment of the household and villages, and modify patterns of daily living, access to affordable non-solid fuel cookstoves has remained a major barrier to reducing solid fuel use [11].

HL is defined by the Institute of Medicine as “the individuals’ capacity to obtain, process and understand basic health information and services needed to make appropriate health decisions” [12]. HL enables one to make informed decisions and to advocate for themselves, their family, and for their community. Persons with lower HL may be less inclined to appreciate the relationship between lifestyle factors and health outcomes, which may suppress constructive health behavior changes [13].

Environmental health literacy (EHL) is a domain of HL that describes the ability to search for, understand, evaluate, and use environmental health information to promote more informed choices and reduce health risks [14, 15]. In this frame, EHL combines theories from both environmental and health literacy to grow the expertise and knowledge needed to both understand and use information that will lead to behavioral changes. Collective improvements in EHL will hopefully lead to positive health effects for individuals as well as for their families and the environment-at-large.

Despite current and expected increased levels of investment in delivery of cleaner cookstove technologies, current uptake has not proven sufficient for ensuring retained use to yield significant public health benefits. We hypothesize that EHL may be associated with health outcomes attributable to HAP, providing further evidence that EHL may be used to help identify barriers to improved cookstove adoption and improve health outcomes.

## Methods

Subjects were randomly selected from households in the community of Kasarani, outside of Nairobi, Kenya. In total, 360 households were approached and asked to participate. The surveys were translated from English to Swahili by a professional interpreter and administered by community health workers affiliated with St. Francis Community Hospital in Kasarani. Answers were given by the household member who responded. Responses were independently back translated from Swahili to English. Seven individuals refused, which led to a 98% participation rate (353/360).

Data was collected using a questionnaire designed to examine the association between HL, EHL and frequency of HAP-associated symptoms [16]. The questionnaire also asked questions related to demographics and cumulative HAP exposure. A number of survey questions were based on earlier validated non-American health literacy surveys [17, 18]. Data included: 1) measures of cooking fuel exposure, 2) household income and education level, 3) variables used as proxies to assess HL and EHL levels, and 4) frequency of HAP-associated symptoms (cough, shortness of breath, phlegm production, wheeze, chest tightness, headache, eye irritation, or burns from cooking). HL assessment included questions regarding the degree to which individuals agreed with the following: comprehension and ability to use information received by physicians to make health decisions, find information on treatment options, and take part in activities that improve health within their community. EHL was assessed using questions such as: 1) breathing smoke from the jiko can lead to coughing, 2) pollution can be caused by smoke from the jiko, and 3) I can’t do much about it, scored using a 5-point Likert scale. Mean HL and EHL were coded using a 5-point scale, with 5 indicating high HL or EHL, and 1 indicating low. The mean response of 11 questions was included in the calculation of mean HL and response of 28 questions was used in the calculation of mean EHL.

Composite variables were created to measure mean EHL, exposure, symptoms, and self-reported health. Fuel type was collapsed into a dichotomous (solid vs non-solid) variable. Non-solid fuels included electricity, liquefied petroleum gas (LPG), biogas, and kerosene. Solid fuels included coal, charcoal, wood, straw, shrubs, agricultural crop, and dung. Mean exposure incorporated type of fuel use, frequency of fuel use, and presence of ventilation. Ten questions regarding household exposure were recoded to 1 (high exposure) or 0 (low exposure) then averaged to calculate the mean exposure rate for each individual. Symptom frequency was reported on a scale of 1–5 and included self-reported cough, shortness of breath, productive cough, wheeze, chest tightness, headache, and eye irritation. Individuals were then classified based on symptom frequency. Individuals who experienced one or more symptoms ≥5 times per month were compared to individuals who experienced symptoms once per month or not at all. Education was dichotomized to none/primary versus secondary/college and income was dichotomized at the median value of 3000KSH. Finally, self-reported health was dichotomized to good/very good/excellent versus fair/poor.

Baseline characteristics were compared by symptom status using a two-sample t-test or chi-square test. Frequency of self-reported symptoms were compared between high HAP exposure and low HAP exposure (dichotomized at the mean of 0.51). Univariate logistic regression was used to assess the association between fuel type, mean exposure, EHL, HL, and self-reported health score with frequency of symptoms at least 5 times per month. Univariate models were then stratified by solid versus non-solid fuel use. Multivariate logistic regression was used to assess the association between symptoms at least 5 times per month and HL, adjusting for EHL, self-reported health (good-excellent vs. poor-fair), and education level. Given suggestion of an interaction between EHL and HL (p for interaction = 0.09), we then stratified our model by median EHL. The model was also stratified by fuel type (solid versus non-solid). Given the potential health risk associated with kerosene use, we ran a sensitivity analysis including only kerosene users. All statistical analyses were conducted using STATA version 16.0.

## Results

**Fig. 1:**
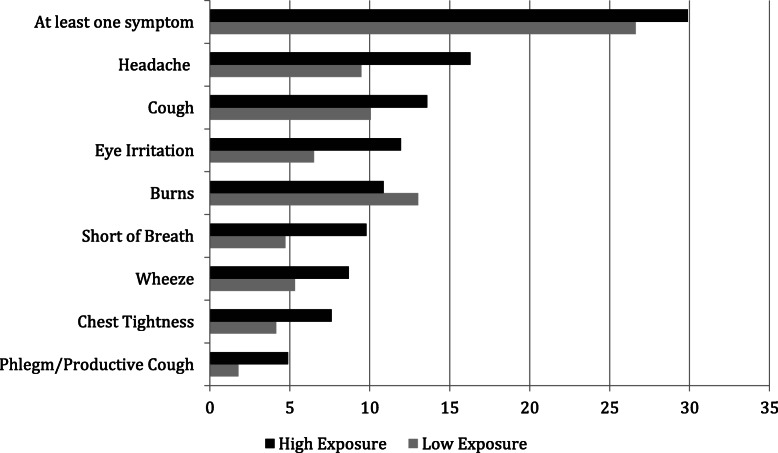
Percent of Individuals Interviewed in Kasarani, Kenya Experiencing One or More Household Air Pollution-Associated Symptoms at Least 5 Times per Month

Among our 353 participants, 28% reported symptoms at least 5 times per month (Table 1). Symptomatic individuals reported similar average exposure to HAP (*p* = 0.41) and clean versus solid fuel use (*p* = 0.89). Health was perceived as fair/poor among 51.3% of symptomatic individuals, compared to 42.3% of asymptomatic individuals (*p* = 0.18). Mean HL, education and income levels were similar among less symptomatic and symptomatic individuals. Individuals who reported high rates of HAP exposure had higher frequency of headache (16.3% versus 9.5%; *p* = 0.06), and shortness of breath (9.8% versus 4.7%; *p* = 0.07) (Fig. 1).

**Table 1:** Characteristics of Individuals Interviewed in Kasarani, Kenya (*n* = 353)

Characteristics^**+**^	Symptom Frequency ≥ 5 per month (***n*** = 100)	Symptom Frequency < 5 per month (***n*** = 253)	*p*-value
Cooking fuel use			0.89
Clean fuel	44 (30.3)	101 (69.7)	
Solid fuel	38 (31.2)	84 (68.9)	
Health perception			0.19
Excellent/very good/good	37 (27.6)	97 (72.4)	
Fair/poor	39 (35.5)	71 (64.6)	
Education level			0.18
None/Primary	37 (25.9)	106 (74.1)	
Secondary/College	55 (32.9)	112 (67.1)	
Household Income/month			0.21
KSH 0–3000	48 (68.8)	106 (31.2)	
KSH 3001-10,000	37 (24.7)	113 (75.3)	
Exposure score*	0.53 ± 0.23	0.51 ± 0.22	0.41
Environmental health literacy score*	3.67 ± 0.40	3.54 ± 0.36	0.002
Health literacy score*	4.39 ± 0.58	4.48 ± 0.67	0.28

^+^N (row %). Missing data on fuel use (*n* = 86), self-perceived health (*n* = 109), and education (*n* = 43)

*Range for composite variables: exposure 0–1, environmental health literacy 1–5, health literacy 1–5. Listed as mean ± standard deviation

In our univariate models, we found that higher HL was associated with lower risk of experiencing symptoms among those with high EHL (OR = 0.53; 95% CI 0.28–1.01), but not among those with low EHL (OR = 0.84; 95% CI 0.52–1.34). After adjusting for education, EHL, and self-reported health we found that higher HL was associated with lower risk of experiencing symptoms (OR = 0.46; 95% CI 0.25–0.86; Table 2). Among those with high EHL, increasing HL was associated with lower risk of experiencing symptoms (OR = 0.26; 95% CI 0.10–0.67), however, there was no association among individuals with low EHL (OR = 0.85; 95% CI 0.34–2.13). Among solid fuel users, the association between HL and risk of experiencing symptoms was driven by individuals with high EHL (OR = 0.30; 95% CI 0.05–1.84), rather than those with low EHL (OR = 1.22; 95% CI 0.36–4.16). Similarly, non-solid fuel users with high EHL had lower risk of experiencing symptoms (OR = 0.22; 95% CI 0.06–0.75), but not among those with low EHL (OR = 0.70; 95% CI 0.13–3.90). In our sensitivity analysis of kerosene users, we found that increasing HL was associated with lower risk of experiencing symptoms among individuals with high EHL (OR = 0.17; 95% CI 0.04–0.77), but not among those with low EHL (OR = 0.86; 95% CI 0.13–5.45). We found a similar association between HL and relative risk of daily increase in symptoms (RR = 0.01, 95% CI 0.00–0.13).

**Table 2:** Association between Health Literacy, Environmental Health Literacy and Household Air Pollution-Associated Symptoms Greater than Weekly Among Solid and Non-Solid Fuel Users (*n* = 172)

Health Literacy*	Odds Ratio	95% Confidence Interval
Solid & Non-Solid Fuel Users (n = 172)		
Solid & Non-Solid Fuel Users^1^	**0.46**	**0.25–0.86**
Environmental Health Literacy^2^		
Low	0.85	0.34–2.13
High	**0.26**	**0.10–0.67**
Solid Fuel Users (*n* = 80)		
All Solid Fuel Users^3^	0.59	0.21–1.64
Environmental Health Literacy^4^		
Low	1.22	0.36–4.16
High	0.30	0.05–1.84
Non-Solid Fuel Users (n = 92)		
All Non-Solid Fuel Users^3^	**0.30**	**0.12–0.76**
Environmental Health Literacy^4^		
Low	0.70	0.13–3.90
High	**0.22**	**0.06–0.75**

*Odds ratios listed for odds of having at least one symptom ≥5 times per month per one unit increase in mean HL. Reference group listed as symptoms < 5 times per month or asymptomatic

^1^Adjusted for environmental health literacy, self-perceived health score, education, and solid fuel use

^2^Model stratified by environmental health literacy adjusted for self-perceived health score, education, and solid fuel use

^3^Modal stratified by solid fuel use adjusted for environmental health literacy, self-perceived health score, and education

^4^Model stratified by environmental health literacy and solid fuel use adjusted for self-perceived health score and education

## Discussion

The Lancet Commission on Pollution and Health recommends cleaner fuels and cookstoves at all levels of intervention [5]. A number of studies examining factors preventing uptake of improved cookstove technologies in resource-limited settings have focused on economic and select sociocultural factors, however, resulting interventions providing access to cleaner cookstoves and non-solid fuel have yielded limited to no uptake of clean cookstoves [19]. Our novel results suggest that higher EHL may help improve HAP-associated health outcomes among individuals who use solid fuel stoves. For example, those with an understanding of the role of smoke from the traditional jiko on health may be more likely to utilize proper ventilation or be willing to switch to a clean cookstove.

We conducted a survey designed with an emphasis on HL and EHL questions administered to an underserved population in East Africa. Although there are validated measures to assess HL levels in high income countries [20], little research has evaluated HL in LMICs, and so there is little information available to evaluate the burden and scope of low HL there [21]. In addition, less is known about EHL and measures have not yet been fully developed. As stated by Ros Dowse, “valid, meaningful data will only be generated if measures reflect local context, culture, and linguistic diversity, accommodate the full range of literacy and cognitive skills, and do not exclude sectors of the population” [22] A number of survey questions, however, were based on earlier validated non-American health literacy surveys [17, 18].”

In an effort to explore the role of EHL and implementation of clean cookstoves, we performed an exploratory analysis of the association between EHL, symptoms, and willingness to change to a clean cookstove. We found that 95.4% of individuals experiencing symptoms at least 5 times per month were willing change their stove type, while this was only true of 76.4% of individuals who experienced fewer symptoms (*p* < 0.001). Given our limited sample of individuals with data on willingness to change stove type among symptomatic solid fuel users, we had limited power to detect an association between EHL and willingness to change stove type (*p* = 0.06). Incorporating EHL into future interventions may be a potential way to address the global burden of HAP in LMICs.

The purpose of EHL is to prevent environmentally induced diseases through intervention and education. It concentrates on addressing the pollution sources and promoting behavior changes to prevent or lessen exposure to pollutants [15]. EHL can be seen as a means that both individuals and groups use for critical reflection within their local socioeconomic setting rather than as a type of HL that integrates specific expertise of environmental factors [23, 24]. Understanding how levels of HL and EHL may influence health behaviors regarding use of solid- and non-solid fuels for cooking could inform future design and implementation of effective public health programs to address HAP.

The Clean Cooking Alliance, an initiative hosted by the United Nations Foundation, has distributed more than 115 million clean cookstoves to households since 2010, with a goal of achieving universal access to clean cookstoves by 2030 [25]. The Alliance has identified Kenya as one of eight priority countries for clean cookstove provision [26]. While this significant investment is important for reducing economic barriers, implementation should be paired with sufficient measures to enable uptake and retention. Our results suggest that improving EHL may improve health outcomes among populations during their transition from solid fuel to clean cookstoves.

To the best of our knowledge, this was the first study to assess the association between EHL, HL and HAP-associated health outcomes. By doing so, we provide researchers and other stakeholders with a better understanding of the importance of EHL in HAP-associated health outcomes in order to develop public health solutions to address HAP from solid fuel use. One strength of our study was our participation rate (98%) which allowed us to enroll a large sample of 353 homes. This success is attributed to our longstanding relationship with the community. Given the limited number of solid fuel users (*n* = 80) and non-solid fuel users (*n* = 92) in our model, however, we may have had insufficient power to detect an association between HL and symptoms among our solid fuel users and high EHL solid fuel users. Additionally, of the 353 individuals, 42 reported cough, 26 reported shortness of breath, 12 reported phlegm, 25 reported wheeze, 21 reported chest pain, 46 reported headache, 33 reported eye irritation, and 42 reported burns ≥5 times per month. Therefore, we had insufficient sample size to evaluate the association between EHL, HL and individual symptoms. Larger studies are needed to further evaluate the role of EHL, HL and specific symptoms associated with HAP. Additional research is needed to assess the determinants of HL and EHL in Kenya and other LMICs. Future research should also specify the household member who should respond, which would allow the study of HAP-associated health outcomes among individuals across the life course, as well as expansion of our study to other communities in LMICs.

## Conclusions

Our findings suggest the importance of promoting EHL among individuals exposed to HAP to improve health outcomes among communities that continue to use solid fuel in Kenya. Future interventions should consider targeting EHL in an attempt to reduce HAP-associated disease.

## Data Availability

The questionnaire used in the current study is available from the corresponding author on reasonable request using the following weblink: www.einstein.yu.edu/faculty/13783/jill-raufman/

## Abbreviations

HAP: household air pollution
LMIC: low- and middle-income country
HL: health literacy
EHL: environmental health literacy
